# On shrinkage and model extrapolation in the evaluation of clinical center performance

**DOI:** 10.1093/biostatistics/kxu019

**Published:** 2014-05-08

**Authors:** Machteld Varewyck, Els Goetghebeur, Marie Eriksson, Stijn Vansteelandt

**Affiliations:** Department of Applied Mathematics, Computer Science and Statistics, Ghent University, 9000 Ghent, Belgium; Department of Statistics, Umeå University, 901 87 Umeå, Sweden; Department of Applied Mathematics, Computer Science and Statistics, Ghent University, 9000 Ghent, Belgium

**Keywords:** Causal inference, Double robustness, Firth correction, Profiling center performance, Propensity score, Quality of care, Random and fixed effects

## Abstract

We consider statistical methods for benchmarking clinical centers based on a dichotomous outcome indicator. Borrowing ideas from the causal inference literature, we aim to reveal how the entire study population would have fared under the current care level of each center. To this end, we evaluate direct standardization based on fixed versus random center effects outcome models that incorporate patient-specific baseline covariates to adjust for differential case-mix. We explore fixed effects (FE) regression with Firth correction and normal mixed effects (ME) regression to maintain convergence in the presence of very small centers. Moreover, we study doubly robust FE regression to avoid outcome model extrapolation. Simulation studies show that shrinkage following standard ME modeling can result in substantial power loss relative to the considered alternatives, especially for small centers. Results are consistent with findings in the analysis of 30-day mortality risk following acute stroke across 90 centers in the Swedish Stroke Register.

## 1. Introduction

In recent years, the interest in profiling hospital performance has grown among different stakeholders including government and health insurers, hospitals and clinicians, and last but not least the patients. Health-care quality thus deserves careful statistical analysis yielding relevant and relatively simple measures with clear interpretation for hospital evaluation.

In this article, we focus on statistical methods to estimate center performance on a binary quality indicator such as 30-day mortality. Causal inference methods will be adopted to adjust for measured confounding by differential patient mix (e.g. initial disease status, age). This is important (DeLong *and others*, 1997; Austin *and others*, 2003) as centers treating more severely ill patients tend to have higher mortality irrespective of treatment quality. Most literature uses indirect standardization to adjust for patient mix (Spiegelhalter, 2005; Shahian and Normand, 2008). This involves contrasting the observed average quality outcome in each center with what it would have been for their patients if “the average level of care over all centers” applied. This is particularly helpful for policy makers when deciding where to best spend resources for quality improvement. However, when centers are expected to provide good health care on the overall patient population, directly standardized outcomes may be of greater interest. This potential full population risk in each center will be our focus. It makes us consider how the entire study population would have fared under the current level of care of each center.

Random effects models which incorporate patient-specific baseline covariates are routinely applied for indirect standardization (Ohlssen *and others*, 2007), and can also be used for direct standardization. The main advantage of these models is that they severely reduce the effective model dimension, thereby avoiding problems of overfitting. However, two main shortcomings deserve more in-depth study: shrinkage and model extrapolation. First, estimates for small centers may shrink severely toward the population mean, resulting in bias and power loss for these centers (Normand *and others*, 1997). This is a major concern because the quality of care in small centers is sometimes questioned, in view of their potentially more limited surgical experience or medical infrastructure (Saposnik *and others*, 2007). Fixed effects (FE) models are no viable substitute in settings encountering many centers with often small numbers of registered patients, where this method suffers from bias and convergence problems (Neyman and Scott, 1948). In this article, we will investigate whether this limitation can be overcome via the Firth correction for fixed center effects models (Firth, 1993). Secondly, when case-mix differs severely between centers, results from the default fixed and random effects models can become very sensitive to model misspecification which is hard to detect (Rubin, 1997). We aim to overcome this using doubly robust methods (Robins *and others*, 2007) that build on a fixed center effects model (with Firth correction) but utilize inverse weighting by the so-called propensity score (PS) (Shahian and Normand, 2008), which is the probability of being treated in the observed center based on patient characteristics.

These statistical methods will be compared in terms of their support for correct detection of low- and more importantly high-risk centers. For this purpose, we will adapt the decision criterion suggested by Normand *and others* (1997) to the framework of direct standardization. Specifically, we will seek solid statistical evidence of a clinically relevant difference between the potential full population risk from a given center and the observed population risk.

Comparisons are made in two case studies: a simulation study on quality insurance for rectal cancer treatment in Belgium and an analysis of quality of care data from the Swedish Stroke Register (Asplund *and others*, 2011). They reflect markedly different settings with chronic versus acute illness, with major versus more limited differences in case-mix, with small versus larger center sizes, and with a limited versus rich set of patient covariates.

## 2. Profiling center performance: framework

Throughout the paper, $C$ is a random variable indicating in which center the patient was actually treated ($C=1, \ldots , m$) and $\textbf {L}$ denotes the vector of patient-specific baseline characteristics such as gender and initial disease status. The methods below focus on 30-day mortality $Y$, but can easily be extended to a continuous or categorical outcome.

### 2.1 Direct versus indirect standardization

Direct standardization aims to infer the potential full population risk for each center $c$: the risk that would be realized if all patients under study were to experience the care level of that given center $c$, irrespective of where they were actually treated. We denote this by $E\{Y(c)\}$, where $Y(c)$ indicates the potential outcome for a given patient if treated at the care level of center $c$. A main feature of direct standardization is that the patient mix used for comparison is a common set of subjects. As such, center comparisons are based on their current performance in the extended patient population, where the extent of extrapolation from each center's own patient population depends on how case-mix differs between centers. This approach may thus evaluate a center's performance based on patients it is not likely to treat.

In contrast, indirect standardization focuses on what a center achieves for its own patient mix. For instance, the frequently used standardized mortality ratio (SMR) takes the ratio of the center's observed risk and the expected risk if these patients would experience the average care level across all centers where center performance levels were uniformly distributed, i.e.

(2.1) \begin{equation*}\label{eq2.1} \mbox{SMR} = \frac{E\{ E(Y|\textbf{L}, C=c) | C=c \}}{m^{-1} \sum_{c^*=1}^m E\{ E(Y|\textbf{L}, C=c^*) |C=c \}} = \frac{E\{Y(c)|C=c\}}{m^{-1} \sum_{c^*=1}^m E\{Y(c^*)|C=c\}} \end{equation*}

 (DeLong *and others*, 1997; Shahian and Normand, 2008). When a difference is taken instead of a ratio, the name “excess risk” is used (Goetghebeur *and others*, 2011). Indirect standardization thus aims to answer the question: “How would the risk in a given center change if its patients were to experience the average risk across all centers?”. Such contrast with the average risk across all centers can be limiting when this reference deviates from what is ideally targeted.

Direct and indirect standardization extrapolate observations to a general population or a general care level, respectively. This may result in different comparisons, as illustrated in Table 1 where centers 1 and 2 have the same patient-specific mortality risks, but differ in patient mix. Following indirect standardization, these centers are classified as having different performance because their patient mix differs. Moreover, results depend on whether indirect standardization is based on SMRs or excess risks, because small absolute differences can result in large relative differences and vice versa. Therefore, when indirectly standardized outcomes are of interest, we would recommend excess risks emphasizing the possibly large extrapolation of center performance. The directly standardized risks on the other hand detect equal quality of care in centers 1 and 2, because of their equal patient-specific mortality risks. They also allow for direct comparison with the overall risk of 7.02%, but may involve serious extrapolation when the patient population of that center differs substantially from the overall population.

**Table 1.** Artificial example comparing center performance based on indirect and direct standardization

	Mortality risk (no. patients)		Indirect standardization		Direct standardization
	$L= {\mathrm {low}}$	$L = {\mathrm {high}}$	SMR	Excess risk	
Center 1	1% (900)	10% (100)	0.8382	$-$0.0037	0.0670
Center 2	1% (100)	10% (900)	0.9349	$-$0.0063	0.0670
Center 3	2% (100)	12% (900)	1.1301	0.0127	0.0833

For the three centers patient-specific mortality risks and patient mix (no. patients) are given per level of the covariate $L$, indicating low or high baseline severity.

### 2.2 Decision criterion for labeling centers

In Section 3, we will compare statistical methods for direct standardization in terms of correctly detecting low- and more importantly high-risk centers. Therefore, following a proposal first introduced in a Bayesian context (Normand *and others*, 1997), we will classify a center as low/high risk if the data provide sufficient evidence that the potential risk $E\{Y(c)\}$ exceeds a benchmark relative to the population average risk $E(Y)$. For this purpose, we will develop estimators $\hat {E}\{Y(c)\}$ for the potential risk $E\{Y(c)\}$ (see Section 3) and then classify a center as low risk if

(2.2) \begin{equation*}\label{eq2.2} \hat{E}\{Y(c)\} + z_k \times \mbox{sd}(\hat{E}\{Y(c)\}) <(1-\lambda) E(Y), \end{equation*}

or as high risk if

(2.3) \begin{equation*}\label{eq2.3} (1+\lambda) E(Y) < \hat{E}\{Y(c)\} - z_k \times \mbox{sd}(\hat{E}\{Y(c)\}). \end{equation*}

Here, $\lambda $ expresses a clinically meaningful tolerance level (e.g. $20\%$) indicating how much the center-specific potential risk is allowed to depart from the current population average risk $E(Y)$. The latter can be estimated by the sample average of observed risks or be replaced by a reference standard if objective benchmarks (e.g. national guidelines) are available. In practice such envisaged reference is likely to steer the choice of $\lambda $ once $E(Y)$ is known or has been estimated. Further, $z_k$ is the $k \times 100$th percentile of the standard normal distribution, so $k$ (e.g. 0.75) expresses the degree of statistical evidence required before flagging a center as low/high risk.

The previous criterion has close links to the often used frequentist criterion (DeLong *and others*, 1997) whereby a center $c$ is classified as low/high risk if the estimated $95\%$ confidence interval for its potential full population risk excludes the population average risk $E(Y)$:

(2.4) \begin{equation*}\label{eq2.4} E(Y) \notin [\hat{E}\{Y(c)\} \pm z_{0.975} \times\mbox{sd}(\hat{E}\{Y(c)\})]. \end{equation*}

See Shahian and Normand (2008) for a related Bayesian criterion. This corresponds with (2.2) and (2.3) if $\lambda =0$ and $k=0.975$. A key drawback of this criterion is that it disregards clinical significance. In particular, large centers are virtually guaranteed to exclude the population average risk and thus will nearly always be labeled statistically significant low-/high-risk centers.

Pure ranking based on the estimated potential risk is dangerous as it is oblivious to a clinical appreciation of differences between centers as well as to uncertainty. Large differences in ranks may correspond with small clinical differences and vice versa. Moreover, uncertainty around the ranking is often substantial (especially for small centers) and confidence intervals may tend to overlap for different centers (Spiegelhalter, 2005). Although ranking based on the estimated probability of exceeding performance can be considered (Normand *and others*, 1997), we believe its inherent property of masking the size of differences in center performance demands careful interpretation involving additional information; it will therefore not be considered in this paper.

## 3. Regression methods

We will now discuss different methods to estimate the potential full population risk $E\{Y(c)\}$ in each center $c=1, \ldots , m$. Let $n$ be the total sample size. Throughout we assume that the patient-specific covariates $\textbf {L}$ are sufficient to adjust for confounding of the center-outcome effect, so that $Y(c) \perp \!\!\!\perp C | \textbf {L}$ for all $c$ (Hernán and Robins, 2006a). Under this assumption, we have that

(3.1) \begin{equation*}\label{eq3.1} E\{Y(c)\} = E \{ E(Y |\textbf{L}, C=c) \}. \end{equation*}

### 3.1 Normal mixed effects model

We will first focus on outcome regression models that postulate that, in each center $c$,

(3.2) \begin{equation*}\label{eq3.2} E(Y|\textbf{L}, C=c) = {\mathrm{expit}} (\textbf{L}'{\boldsymbol \beta} + \psi_c)= f(\textbf{L}, c; {\boldsymbol \beta}, {\boldsymbol \psi}), \end{equation*}

where ${\boldsymbol \psi } = (\psi _1, \ldots , \psi _m)$ are the center effects. For convenience, we here constrain the effects ${\boldsymbol \beta }$ of patient-specific covariates on outcome to be equal across all centers, but this can in principle be checked (size permitting) or relaxed by including interactions with center. Once estimates $(\hat {\boldsymbol \beta }, \hat {\boldsymbol \psi })$ for $({\boldsymbol \beta }, {\boldsymbol \psi })$ have been obtained, it follows from (3.1) that the potential full population risk can be estimated as follows (Hernán and Robins, 2006a):

(3.3) \begin{equation*}\label{eq3.3} \hat{E}\{Y(c)\} = \frac{1}{n} \sum_{i=1}^n {\mathrm{expit}} (\textbf{L}_i'\hat{\boldsymbol \beta} + \hat{\psi}_c) = \frac{1}{n} \sum_{i=1}^n f(\textbf{L}_i, c; \hat{\boldsymbol \beta}, \hat{\boldsymbol \psi}). \end{equation*}

The evaluation of center performance is often based on Bayesian normal mixed effects (ME) models. These augment model (3.2) with a normal random effects distribution

(3.4) \begin{equation*}\label{eq3.4} \psi_c \sim N(\mu_{\psi}, \sigma_{\psi}^2), \quad c=1,\ldots, m, \end{equation*}

which assumes centers to be exchangeable in the sense that any *a priori* information on the relative ordering or grouping of center effects is ignored (Ohlssen *and others*, 2007). Here, $\mu _{\psi }$ is the common mean and $\sigma _{\psi }$ is the standard deviation of the center effects $\psi _c$, which we assume to have independent hyperpriors. Moreover, assuming that the center effects are *a priori* independent of the effects of patient characteristics, the joint posterior distribution of this two-level Bayesian approach is of the form

(3.5) \begin{equation*}\label{eq3.5} p(\boldsymbol{\beta}, \boldsymbol{\psi}, \mu_{\psi}, \sigma^2_{\psi}|\textbf{y}, \textbf{L}, \textbf{C}) \propto \prod_{i=1}^n p(y_i|\boldsymbol{\beta}, \boldsymbol{\psi}, \textbf{L}, \textbf{C}) p(\boldsymbol{\psi}|\mu_{\psi}, \sigma^2_{\psi}) p(\boldsymbol{\beta}) p(\mu_{\psi}) p(\sigma^2_{\psi}). \end{equation*}

This posterior is estimated using a Markov chain Monte Carlo (MCMC) algorithm, which provides values for the model parameters $({\boldsymbol \beta }, {\boldsymbol \psi })$ in each step. These are subsequently used to evaluate (3.3), thereby enabling us to estimate the posterior distribution of $E\{Y(c)\}$.

An advantage of the Bayesian over a frequentist approach based on empirical best linear unbiased predictions (Robinson, 1991) is that, by using MCMC algorithms, one can directly obtain posterior estimates and variances of even complicated transformations such as (3.3) without the need for large sample justifications, provided a sufficient number of MCMC iterations are run (O’Brien and Dunson, 2004). Prior information can be incorporated in Bayesian models through an informative prior distribution. When no such information is available, a normal distribution with large variance as non-informative prior on center level may still be hard to justify as the center effects could follow a longer-tailed distribution such as the Student's $t$ or even an asymmetric distribution. In particular, choosing a normal prior may shrink estimated center effects toward the center population mean $\mu _{\psi }$, especially for very small centers (Normand *and others*, 1997). Severe shrinkage may be problematic when reporting individual feedback to the centers, because identification of centers with deviating performance is especially important. The amount of shrinkage is related to the choice of prior, but judging the plausibility of a normal prior is difficult because it refers to center effects on the logit scale.

### 3.2 Reducing shrinkage

#### 3.2.1 Clustered normal ME model

The mixture model of Ohlssen *and others* (2007) forms a first approach considered to reduce shrinkage. This involves assigning the $m$ centers to a chosen number $K<m$ of clusters each with their own normal random effects distribution. The model for the center level effects thus becomes

\[ \psi_c \sim N(\mu_k, \sigma_k^2) \quad \mbox{with unknown probability } p_k, \quad k=1, \ldots, K. \]

It thus assigns each center $c$ to cluster $k$ with probability $p_k$ and subsequently draws a random center effect from the normal distribution of the “cluster $k$ population” with cluster mean $\mu _k$ and variance $\sigma _k^2$ within the cluster. In this process, we let each center have an *a priori* equal probability of belonging to each cluster without the size or performance of the clusters being predefined. When *a priori* knowledge of clustered center performance is available (e.g. when large centers are expected to have better facilities resulting in better performance (Saposnik *and others*, 2007)), it can be incorporated by giving centers a larger prior probability for specific clusters.

#### 3.2.2 FE logistic regression model with Firth correction

Shrinkage can alternatively be reduced using maximum likelihood estimation for the FE logistic regression model (3.2). However, because of overfitting the resulting estimator (3.3) may behave erratically when there are centers with few events: besides convergence problems, there may be substantial finite sample bias and large variance (Peduzzi *and others*, 1996). The ME approach of Section 3.1 accommodated this by imposing a normal distribution on the center effects. Here, we will consider the Firth corrected FE method instead.

Firth correction (Firth, 1993) reduces the $O(n^{-1})$ bias of ordinary maximum likelihood estimators to the order $O(n^{-2})$ by maximizing the penalized likelihood function

(3.6) \begin{equation*}\label{eq3.6} L^*({\boldsymbol \beta}, {\boldsymbol \psi}) = L({\boldsymbol\beta}, {\boldsymbol \psi}) |I({\boldsymbol \beta}, {\boldsymbol\psi})|^{1/2}, \end{equation*}

instead. Here, $|I(\cdot)|$ denotes the determinant of the Fisher information matrix of $({\boldsymbol \beta }, {\boldsymbol \psi })$ and $L(\cdot)$ is the ordinary likelihood function. Since $|I({\boldsymbol \beta }, {\boldsymbol \psi })|^{1/2}$ equals Jeffreys’ invariant prior, Firth correction is equivalent to penalization of the likelihood by Jeffreys’ prior (Kosmidis and Firth, 2009), suggesting that Firth corrected maximum likelihood estimates are also subject to shrinkage. However, Jeffreys’ prior is invariant under reparameterization and has the key feature of being non-informative. The latter, coupled with its defining bias reduction property, implies that it may result in less shrinkage compared with the use of a normal prior. There is evidence that it may also perform better in terms of other properties such as finiteness of the estimator and coverage of confidence intervals (Kosmidis and Firth, 2009). We will investigate this in our setting through simulations in Section 4. In supplementary material available at *Biostatistics* online (Sections 1.1 and 1.2), we give additional detail on the Firth correction and show how to estimate the asymptotic variance of the resulting estimate of $E\{Y(c)\}$.

### 3.3 Accounting for model extrapolation: doubly robust PS method

All previous methods suffer from a risk of extrapolation as they require predicting for each patient how he/she would fare if the care level of a given center $c$ applied. When case-mix differs across centers, especially when there are strong confounders, such extrapolation may not be justified. Even models that seem to fit the observed data well may then be misspecified and imply serious model extrapolation, resulting in bias and underestimated uncertainty (Rubin, 1997). This is illustrated in supplementary material available at *Biostatistics* online (Figure 3) where we consider two centers with strongly differential case-mix: one center has patients older than 60 and the other does not. We find strong model extrapolation that may not get reflected in standard errors, so the user is left without warning (Rubin, 1997). Similar concerns are warranted for standard indirect standardization methods as these extrapolate stratum-specific center effects to the patients of each given center.

Inverse probability weighting via PS avoids extrapolation by not relying on outcome models. For a given patient $i,$ the PS are defined as the vector of probabilities to belong to each given center $c$ on the basis of his/her baseline characteristics $\textbf {L}_i$. In practice, such PS can be estimated by fitting a multinomial regression model:

(3.7) \begin{equation*}\label{eq3.7} P(C_i = c|\textbf{L}_i) = g(\textbf{L}_i, c; {\boldsymbol \gamma}, {\boldsymbol \delta}) = \begin{cases} \dfrac{1}{1+ \sum_{j=2}^m \exp(\textbf{L}'_i {\boldsymbol \delta}_j + \gamma_j)}, & c = 1, \\ \dfrac{\exp(\textbf{L}'_i {\boldsymbol \delta}_c + \gamma_c)}{1+ \sum_{j=2}^m \exp(\textbf{L}'_i {\boldsymbol \delta}_j + \gamma_j)}, & c \neq 1, \end{cases} \end{equation*}

where $C_i$ indicates the center where patient $i$ was treated. Parameter estimators $(\hat {\boldsymbol \gamma },\hat {\boldsymbol \delta })$ can be obtained via maximum likelihood, so by solving the following set of estimating equations:

(3.8) \begin{equation*}\label{eq3.8} \frac{1}{n} \sum_{i=1}^n \left(\begin{matrix} 1 \\ \textbf{L}_i \end{matrix} \right) \{ I(C_i=c) - g(\textbf{L}_i, c; \hat{\boldsymbol \gamma}, \hat{\boldsymbol \delta})\} = \textbf{0}, \quad c =2, \ldots, m. \end{equation*}

We can now estimate $E\{Y(c)\}$ as in (3.3), but using a weighted regression to fit the FE model (3.2) with weights equal to 1 over the PS of the observed center $g(\textbf {L}_i, C_i; \hat {\boldsymbol \gamma }, \hat {\boldsymbol \delta })$. That this works can be seen because the weighted regression of the FE model sets

(3.9) \begin{equation*}\label{eq3.9} \frac{1}{n} \sum_{i=1}^n \left(\begin{matrix} \textbf{L}_i \\ I(C_i = 1) \\ \vdots \\ I(C_i = m) \\ \end{matrix}\right) \frac{1}{g(\textbf{L}_i, C_i; \hat{\boldsymbol \gamma}, \hat{\boldsymbol \delta})} \{ Y_i - f(\textbf{L}_i, C_i; \hat{\boldsymbol \beta}, \hat{\boldsymbol \psi})\} = \textbf{0}. \end{equation*}

This enables us to rewrite

(3.10) \begin{align} \hat{E}\{Y(c)\} &= \frac{1}{n} \sum_{i=1}^n f(\textbf{L}_i, c; \hat{\boldsymbol \beta}, \hat{\boldsymbol \psi}) \nonumber \\ &= \frac{1}{n} \sum_{i=1}^n \left[ f(\textbf{L}_i, c; \hat{\boldsymbol \beta}, \hat{\boldsymbol \psi}) + \frac{I(C_i=c)}{g(\textbf{L}_i, c; \hat{\boldsymbol \gamma}, \hat{\boldsymbol \delta})} \{ Y_i - f(\textbf{L}_i, c; \hat{\boldsymbol \beta}, \hat{\boldsymbol \psi})\} \right] \label{eq3.10}\end{align}

(3.11) \begin{equation*} = \frac{1}{n} \sum_{i=1}^n \left[ \frac{I(C_i=c) Y_i}{g(\textbf{L}_i, c; \hat{\boldsymbol \gamma}, \hat{\boldsymbol \delta})} + \left\{ 1 - \frac{I(C_i=c)}{g(\textbf{L}_i, c; \hat{\boldsymbol \gamma}, \hat{\boldsymbol \delta})} \right\} f(\textbf{L}_i, c; \hat{\boldsymbol \beta}, \hat{\boldsymbol \psi}) \right].\label{eq3.11} \end{equation*}

These expressions show how the resulting estimator of $E\{Y(c)\}$ is doubly robust, i.e. unbiased (in large samples) if either the outcome or the PS model holds, but not necessarily both (Robins *and others*, 2007). Indeed, the second term in (3.10) has population mean zero if the outcome model is correctly specified and the second term in (3.11) for a correct PS model. Furthermore, the remaining term has mean $E\{Y(c)\}$ under those respective assumptions. This double robustness property is attractive because it allows for misspecification of the FE model if the PS is modeled correctly. It thus, in particular, offers partial protection against false omission of interactions between center and patient characteristics. When patient mix is drastically different between centers, the resulting lack of information gets exhibited in large standard errors.

Small centers can give problematically small estimated PS values, especially when its patient case-mix is very different from that of other large centers. We therefore stabilize the PS for each center by dividing all estimates $g(\textbf {L}_i, c; \hat {\boldsymbol \gamma }, \hat {\boldsymbol \delta })$ by the proportion of patients at that center. This stabilization does not affect the consistency nor the double robustness property of $\hat {E}\{Y(c)\}$. These properties are also not affected by applying the Firth correction when fitting the outcome model (3.2) by weighted regression, because this is a finite-sample correction. In supplementary material available at *Biostatistics* online (Section 1.3), we give details on how the asymptotic variance of $\hat {E}\{Y(c)\}$ is estimated.

## 4. Results

### 4.1 Simulation study application: Belgian colorectal cancer register

The Belgian cancer register collected data on colorectal cancer diagnosis and follow-up between 2006 and 2010. This fairly young register with voluntary participation has a national coverage of about $30\%$. We will consider a total of 2355 patients treated in 63 centers and examine a binary outcome quality indicator with $22\%$ events on average. Causal inference methods on this dataset were introduced by Goetghebeur *and others* (2011), with descriptive statistics showing substantial heterogeneity in case-mix.

**Fig. 1.**
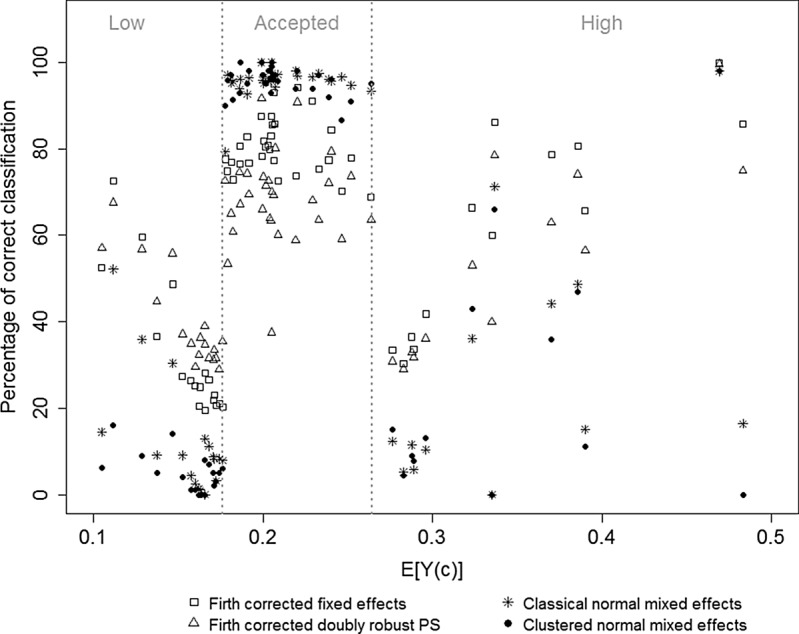
Percentage of correct classification against true potential full population risk for each center and regression method, based on the simulation study mimicking the setting of the Belgian colorectal cancer register. The vertical gray lines indicate the clinical decision limits $(1-\lambda) E(Y)$ and $(1+\lambda) E(Y)$, with $\lambda = 0.20$.

We will compare the performance of the normal ME, clustered normal ME, Firth corrected FE, and doubly robust PS methods (Section 3) via simulation experiments that reflect the structure of these data. Comparisons are based on the power and type I error of center classification following the profiling technique described in Section 2.2 with $\lambda =20\%$ and $k=0.75$. For example, the Power to detect High is the probability of classifying a center as having high risk, given that its “true” classification is high. The balance between type I error and power is determined by the values of the clinical ($\lambda $) and statistical ($k$) tolerance levels and the distribution of true alternatives, which are fixed here. Details on the simulation study are given in supplementary material available at *Biostatistics* online (Section 2.1). Results are shown in Table 2 and Figure 1, where it can be seen that the power to detect low-risk centers is generally smaller than the power to detect high-risk centers. This is expected because of the lower number of events and closeness to the boundary for low mortality risk. Because of shrinkage, the normal ME method has very low power and appears unable to detect many of the low-/high-risk centers. Mistakenly classifying centers as low/high mortality risk is equally rare for these methods.

**Table 2.** Center classification $($low/high risk or accepted$)$ based on $1000$ simulations for each regression method

	Classical normal ME	Clustered normal ME	Firth corrected FE	Firth corrected doubly robust PS
Power ($\%$)				
To detect high	28.8	31.2	61.5	53.9
To detect low	12.3	5.4	32.0	39.9
Type I error ($\%$)				
Low as high	0.1	0.3	2.4	6.3
Accepted as high	1.2	2.0	8.6	11.8
Type I error ($\%$)				
High as low	0.1	0.1	0.9	2.9
Accepted as low	3.2	2.1	11.2	19.1
Coverage of $95\%$ CI				
For $E\{Y(c)\}$ ($\%$)	95.4	87.5	93.9	89.0
Classification				
$\%$ high ($22\%$)	7.2	8.0	16.5	17.9
$\%$ low ($30\%$)	6.0	3.0	16.0	22.2
$\%$ correct (L-A-H)	59.6	57.7	62.4	58.1

The true percentage of low- and high-risk centers is, respectively, $30\%$ and $22\%$.

Interestingly, the clustered normal ME method is not performing better. This blind clustering, i.e. irrespective of center characteristics, thus requires considerable computing effort with no payback at the considered sample size. While similar in terms of power, the doubly robust PS method makes more Type I errors than the FE method because of the lower coverage of the nevertheless on average wider confidence intervals. Although the doubly robust PS method has potential value in realistic settings with strong confounding because of its robustness against model misspecification, confidence intervals with better finite sample performance are needed before routine application can be recommended. The percentage of centers that are correctly classified is similar for all methods. For the ME methods, unlike for the other methods, this is the result of classifying nearly all centers as “accepted” (see Figure 1).

In supplementary material available at *Biostatistics* online (Section 2.1), we provide additional simulation results: Applying the Firth correction did not severely influence the results of the uncorrected FE methods, but ensured convergence in the presence of very small centers. Doubling the sample size especially increased the accuracy of the ME methods. When only one (outcome or PS) model is misspecified, we found some evidence favoring the doubly robust PS method over the FE method.

### 4.2 Analysis of the Swedish Stroke Register

Riks-Stroke (http://www.riks-stroke.org) is a national quality register for acute stroke, collecting data from all 90 Swedish hospitals. It has an estimated coverage of the total stroke population between $80\%$ and $90\%$, but there is considerable variation in coverage between hospitals. The setting is conceptually different from the cancer register, since acute stroke is treated urgently, mostly at the nearest center. The register is linked with a socio-economic database at Statistics Sweden and contains 149 778 patients with first stroke between 2001 and 2009. Centers are compared on 30-day mortality, applying the classical normal ME, the FE and the doubly robust PS method, without Firth correction as centers are no longer problematically small.

Because of convergence issues with multinomial models, we build a separate logistic regression model $P(C=c|\textbf {L}) = {\mathrm {expit}}(\textbf {L}' \boldsymbol {\delta }_c + \gamma _c)$ per center $c$ and estimate the PS for individual $i$ treated at center $c$ as follows:

(4.1) \begin{equation*}\label{eq4.1} \frac{P(C_i=c|\textbf{L}_i)}{\sum_{j=1}^m P(C_i=j|\textbf{L}_i)} = \frac{{\mathrm{expit}}(\textbf{L}_i' \boldsymbol{\delta}_c + \gamma_c)}{\sum_{j=1}^m {\rm expit}(\textbf{L}_i' \boldsymbol{\delta}_j + \gamma_j)}. \end{equation*}

The classical ME method is applied separately to the cluster of small ($<$1000 patients), medium (1000–2000) and large ($>$2000) centers, to reduce the effects of shrinkage and to avoid convergence problems due to the large data size. The potential full population risk for this method is then based on the cluster-specific parameter estimates applied to the total population.

While, in general, few data are missing, records on education and smoking are missing for, respectively, $20.8\%$ and $12.8\%$ of the participants. Certain patient characteristics, like smoking status, are more likely missing for patients who were unconscious upon admission and education level is more often unknown for elderly patients. We fit complete-case regression models as they allow for missingness in the covariates to depend on the covariates themselves, so long as there is no residual dependence on the outcome (White and Carlin, 2010); it moreover avoids the need for modeling the distribution of those covariates. They will therefore return center effects that are unbiased under fairly minimal assumptions. Since the proposed estimators standardize these effects to the same reference population, selective missingness is not expected to distort comparison of $E\{Y(c)\}$ between centers, although it may yield underestimates of $E\{Y(c)\}$ for each center, as suggested by the comparison of complete cases ($n= 101\,051$) versus all cases in supplementary material available at *Biostatistics* online (Section 2.2). Supplementary material available at *Biostatistics* online further specifies the covariates that were included in the PS and outcome regression models. It shows that in general case-mix does not differ much across centers, except for considerable variation with respect to treatment for high blood pressure, education level, and time of admission.

**Fig. 2.**
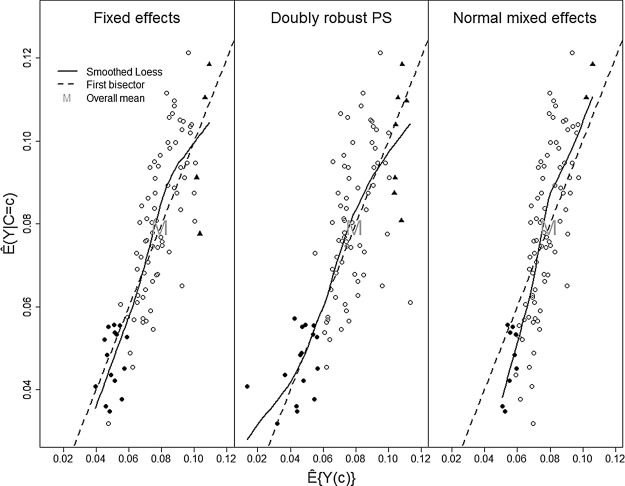
The observed center-specific risk versus the potential full population risk for all 90 centers of Riks-Stroke for the FE method, the doubly robust PS method, and the classical normal ME method, distinguishing between low (filled circle) and high (filled triangle) mortality risk.

**Fig. 3.**
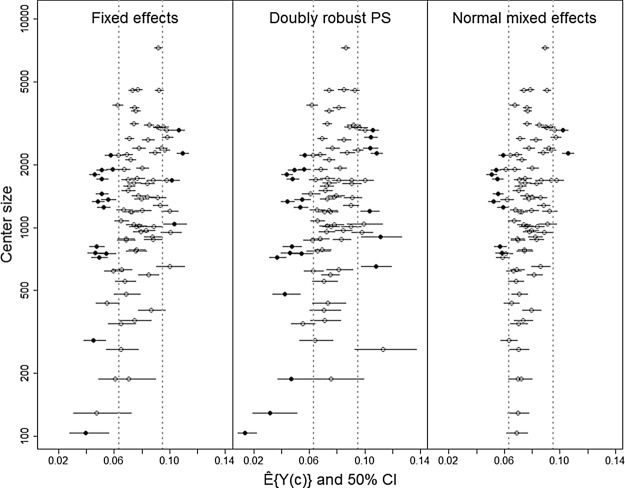
The potential full population risk and corresponding $50\%$ confidence interval for all 90 centers of Riks-Stroke for the FE method, the doubly robust PS method, and the classical normal ME method. The vertical gray lines indicate the clinical decision limits $(1-\lambda) \hat {E}(Y)$ and $(1+\lambda) \hat {E}(Y)$, with $\lambda =0.20$, which are used for classification of low and high (filled circles) mortality risk centers.

The fewer centers classified as low/high risk by the normal ME method are also found by the other methods. This can be seen in Figure 2, which displays the observed center-specific risk $\hat {E}(Y|C=c)$ (which does not depend on the analysis method) versus the model-based potential full population risk $\hat {E}\{Y(c)\}$. The ME results show the smallest range of potential full population risk with most attenuation toward the overall risk, i.e. more shrinkage. Both FE methods classify mostly the same centers as low risk, although the doubly robust PS method classifies two additional centers as high risk (Figure 2). Because these two centers may be only borderline statistically significant, we examine the uncertainty in terms of a $50\%$ CI on the potential full population risk (Figure 3). We found good discrimination of low and high mortality risk in general, but the $50\%$ CI of some centers is close to the clinical decision limit where it becomes difficult to judge. In that case, balanced testing could help to find an optimal combination of the null and the alternative (Moerkerke and Goetghebeur, 2006). Figure 3 shows better accuracy of $\hat {E}\{Y(c)\}$ with increasing center size, except for centers with close to zero events. Surprisingly, high mortality risks are observed mostly for medium to large centers while low mortality risks are especially detected for small to medium centers. This may partly be due to selectivity as it is known that patients dying early are less likely to be recorded in this Riks-Stroke register, which could potentially happen more frequently in smaller centers. Since size is based on the number of registered patients, a center with low coverage may come out as smaller with better performance. This underlines again the importance of complete coverage.

For the doubly robust method, we observe wider confidence intervals especially for the small centers. This may be related to the generally lower efficiency of this method, but also be more honestly reflecting the uncertainty on the potential risk estimates. Unlike the other two methods, the ME method did not classify any of the very small centers as low risk. This is due to shrinkage to which especially the smallest centers are very sensitive.

## 5. Discussion

We have proposed and compared approaches to evaluate the performance of clinical centers via direct standardization. This involves comparing centers in terms of the potential risk if the full study population were treated at the current level of care of the given center. A key feature is that the evaluation of all centers is based on the same reference population, while each center will have treated a subset. Especially when centers can be chosen freely, this cuts bias out of the current center performance. Alternatively, indirect standardization, which is more widely used (Austin *and others*, 2003; Shahian and Normand, 2008), evaluates each center on its own patient population and is of particular relevance when centers tend to differ in their patient mix. Both standardizations have their virtues and in future work we will develop similar analysis strategies for indirect standardization.

We have compared three statistical regression methods for direct standardization based on random or fixed center effects, the latter in combination with the Firth correction or weighting by the reciprocal of the PS to be treated in the observed center. Our primary focus on frequentist methods was motivated by the fact that they are less computer-intensive and avoid the need to specify prior distributions about which no information was available in our case studies. A crucial and unverifiable assumption for all considered methods is that the included set of baseline patient characteristics is sufficient to adjust for confounding of the center-outcome effect. This drives the variable selection at the design stage of disease registers. In case of violations, results will be biased and one may want to consider other methods such as those using instrumental variables (Hernań and Robins, 2006b).

In the first case study, we found that shrinkage following traditional ME modeling results in substantial power loss compared with the suggested alternatives, especially for small centers. Although we used direct standardization, these findings correspond to those observed for indirect standardization in Austin *and others* (2003). The Firth corrected FE model as well as the doubly robust PS method recovered power, while maintaining convergence in the presence of very small centers. In the second case study, shrinkage was still present under the normal ME model, but disappeared using the Firth correction (see supplementary material available at *Biostatistics* online, Section 2.2). As a result, fewer centers were classified as low/high risk under the random compared with the fixed center effects models.

In the simulation study, the Firth corrected FE method outperformed the doubly robust PS method, although differences were relatively minor. While routine application of the doubly robust PS method in its current form is not recommended, it may be of potential interest in settings with strongly differential case-mix (Shahian and Normand, 2008). In such settings, standard variable selection procedures for outcome regression models which force the center effects into the model have a tendency, as a result of multicollinearity, to exclude patient characteristics that are strongly correlated with center choice so that their effects get attributed to differences between centers. This may in turn yield model extrapolation with biased and misleadingly precise center effect estimates (Vansteelandt *and others*, 2010). The doubly robust PS method helps to protect against this. By also modeling the effect of patient characteristics on the center choice, strong predictors of center choice are potentially more likely to be picked up in variable selection procedures (Imbens, 2000). In future work, it will therefore be of interest to evaluate how the considered methods perform when combined with variable selection. Double robustness moreover protects against misspecification of the outcome model when the model for center choice is correct. It thereby lessens the concern for violation of the assumption of equal covariate effects across centers.

## Supplementary material

Supplementary Material is available at http://biostatistics.oxfordjournals.org.

## Funding

This work was supported by the Institute for the Promotion of Innovation through Science and Technology in Flanders (IWT-Vlaanderen) [to M.V.]; IAP research network from the Belgian government (Belgian Science Policy) [grant no. P07/06 to E.G. and S.V.], and the Swedish Research Council [grant no. 2012-5934 to E.G. and M.E.]. Funding to pay the Open Access publication charges for this article was provided by the Swedish Research Council.

## Supplementary Material

Supplementary Data
