# Supplementary material for: On shrinkage and model extrapolation in the evaluation of clinical center performance
Source: Biostatistics. 2014 May 8;15(4):651–64. doi: 10.1093/biostatistics/kxu019 (PMC4173104; doi:10.1093/biostatistics/kxu019)
Supplement: Supplementary Data [file supp_15_4_651__index.html]

On shrinkage and model extrapolation in the evaluation of clinical center performance — Supplementary Data 

# On shrinkage and model extrapolation in the evaluation of clinical center performance

## Supplementary Data

Supplementary Data

**Files in this Supplementary Material:**

- Supplementary Data - Pdf file
