# Supplementary material for: On shrinkage and model extrapolation in the evaluation of clinical center performance
Source: Biostatistics. 2014 May 8;15(4):651–64. doi: 10.1093/biostatistics/kxu019 (PMC4173104; doi:10.1093/biostatistics/kxu019)
Supplement: Supplementary Data [file supp_kxu019_kxu019supp_data.pdf]

Biostatistics (2014), **0**, 0, *pp.* 1–22
doi:10.1093/biostatistics/ComparingMethods-Supplementary-review

On shrinkage and model extrapolation in statistical methods for assessing clinical center performance - Supplementary Material

MACHTELD VAREWYCK*[,1], ELS GOETGHEBEUR[1], MARIE ERIKSSON[2], STIJN VANSTEELANDT[1]

[1] *Department of Applied Mathematics, Computer Science and Statistics, Ghent University, 9000 Ghent, Belgium*

[2] *Department of Statistics, Umeå University, 901 87 Umeå, Sweden*

machteld.varewyck@ugent.be

1. Technical appendices

1.1 *Firth correction*

Maximum likelihood estimation for the parameter $\boldsymbol{\theta} := (\boldsymbol{\beta}, \boldsymbol{\psi})$ indexing a logistic regression model amounts to solving the equations

$$\frac{\partial \ell(\boldsymbol{\theta})}{\partial \boldsymbol{\theta}} = u(\boldsymbol{\theta}) = \mathbf{0}, \tag{1.1}$$

where $\ell(\boldsymbol{\theta}) = \log L(\boldsymbol{\theta})$ is the log likelihood function and $u(\boldsymbol{\theta})$ is the score function for a single observation. Firth (1993) defines a modified score function

$$u^*(\boldsymbol{\theta}) = u(\boldsymbol{\theta}) - I(\boldsymbol{\theta}) b(\boldsymbol{\theta}), \tag{1.2}$$

where $I(\boldsymbol{\theta}) = -\frac{\partial u(\boldsymbol{\theta})}{\partial \boldsymbol{\theta}}$ is the Fisher information matrix for a single observation and $b(\boldsymbol{\theta})$ is the $O(n^{-1})$ bias of the resulting maximum likelihood estimator $\hat{\boldsymbol{\theta}}$. Therefore, the solution θ_F to $u^*(\boldsymbol{\theta}) = \mathbf{0}$ removes the $O(n^{-1})$ bias of $\hat{\boldsymbol{\theta}}$.

A simple interpretation of equation (1.2) is that it specifies an amount of bias to be introduced into the score function in order to remove the leading term in the asymptotic bias of $\hat{\boldsymbol{\theta}}$. The introduced bias function $-I(\boldsymbol{\theta})b(\boldsymbol{\theta})$ is $O(1)$ as $n \to \infty$, an order of magnitude smaller in probability than $u(\boldsymbol{\theta})$ itself (Firth, 1992). The Firth approach eliminates the $O(n^{-1})$ bias of the maximum likelihood estimate by adjusting the score function, rather than the estimate itself. An advantage is that the definition of the Firth corrected estimates does not depend on the existence of the non-corrected estimates, of which some components may be infinite in binomial problems (Firth, 1992).

Simulation studies in different settings for logistic regression in Heinze (2006) show promising results for the Firth method in case of separated or nearly separated data. Based on simulations in Kessels *and others* (2013), the Firth corrected multinomial logistic regression outperforms

*To whom correspondence should be addressed.

© The Author 2014. Published by Oxford University Press. All rights reserved. For permissions, please e-mail: journals.permissions@oup.com

ordinary maximum likelihood as the Firth corrected method converges in case of data separation, it removes bias of the ML estimates and reduces variance.

1.2 *Fixed effects logistic regression: Asymptotic variance*

To simplify notation we denote the vector $(\mathbf{L}, I(C=1), \ldots, I(C=m))$ by $\mathbf{X}$. To assess the uncertainty on the potential full population risk $\hat{E}\{Y(c)\}$, note that the MLE $\hat{\boldsymbol{\theta}} = (\hat{\boldsymbol{\beta}}', \hat{\boldsymbol{\psi}}')'$ of $\boldsymbol{\theta}$ in

$$E(Y|\mathbf{X}) = U(\boldsymbol{\theta}, \mathbf{X}) = \text{expit}\left(\mathbf{L}'\boldsymbol{\beta} + \sum_{c=1}^{m} \psi_c \, I(C=c)\right), \tag{1.3}$$

satisfies the following set of estimating equations

$$\frac{1}{n}\sum_{i=1}^{n} \mathbf{X}_i \left\{Y_i - U(\hat{\boldsymbol{\theta}}, \mathbf{X}_i)\right\} = \mathbf{0}. \tag{1.4}$$

To obtain the asymptotic variance of $\hat{E}\{Y(c)\}$, we first perform a Taylor expansion of

$$\hat{E}\{Y(c)\} = \frac{1}{n}\sum_{i=1}^{n} U_c(\hat{\boldsymbol{\theta}}, \mathbf{X}_i) = \frac{1}{n}\sum_{i=1}^{n} \text{expit}\left(\mathbf{L}_i'\hat{\boldsymbol{\beta}} + \hat{\psi}_c\right) \tag{1.5}$$

around $\boldsymbol{\theta}$, which results in

$$\frac{1}{\sqrt{n}}\sum_{i=1}^{n} U_c(\hat{\boldsymbol{\theta}}, \mathbf{X}_i) = \frac{1}{\sqrt{n}}\sum_{i=1}^{n} U_c(\boldsymbol{\theta}, \mathbf{X}_i) + E\left(\frac{\partial U_c}{\partial \boldsymbol{\theta}}(\boldsymbol{\theta}, \mathbf{X}_i)\right)\sqrt{n}(\hat{\boldsymbol{\theta}} - \boldsymbol{\theta}) + o_p(1). \tag{1.6}$$

Second, we perform a Taylor expansion of (1.4) around the true parameter value $\boldsymbol{\theta}$ and obtain

$$\sqrt{n}(\hat{\boldsymbol{\theta}} - \boldsymbol{\theta}) = \frac{1}{\sqrt{n}}\sum_{i=1}^{n} A^{-1} R_i + o_p(1), \tag{1.7}$$

where

$$R_i = \mathbf{X}_i \left\{Y_i - U(\hat{\boldsymbol{\theta}}, \mathbf{X}_i)\right\},$$
$$A = E\left(\frac{\partial R_i}{\partial \boldsymbol{\theta}}\right).$$

Finally, we combine (1.7) and (1.6) and estimate the variance of $\hat{E}\{Y(c)\}$ via

$$\text{Var}\left[\hat{E}\{Y(c)\}\right] = \frac{1}{n}\text{Var}\left\{U_c(\boldsymbol{\theta}, \mathbf{X}_i) + E\left(\frac{\partial U_c}{\partial \boldsymbol{\theta}}(\boldsymbol{\theta}, \mathbf{X}_i)\right) A^{-1} R_i\right\}. \tag{1.8}$$

Confidence interval calculations assume normality of logit $\hat{E}\{Y(c)\}$, so in our calculations we have transformed these estimators to the logit scale via the delta method. This allows us to obtain confidence intervals with boundaries inside the $[0, 1]$ interval.

The above expressions continue to hold when the parameters $\boldsymbol{\theta}$ are estimated according to Firth's modified estimating equations (1.2). This is because bias reduction affects the covariance matrix of the estimates only in the $O(n^{-2})$ and higher order terms (Firth, 1993). Note that standard errors of the Firth corrected estimates may be slightly smaller, since they are evaluated in a different estimate for $\boldsymbol{\theta}$ (Firth, 1992).

1.3 *Doubly robust PS method: Asymptotic variance*

We denote the outcome model parameters by $\boldsymbol{\theta} = (\boldsymbol{\beta}', \boldsymbol{\psi}')'$ and the propensity score parameters by $\boldsymbol{\rho} = (\boldsymbol{\gamma}', \boldsymbol{\delta}')'$. We estimate $E\{Y(c)\}$ again as in (1.5), but now using a weighted regression to fit the FE model (1.3), with weights equal to one over the propensity score of the observed center $g(C_i, \mathbf{L}_i; \hat{\boldsymbol{\rho}})$, where

$$g(c, \mathbf{L}_i; \boldsymbol{\rho}) := P(C_i = c|\mathbf{L}_i) = \begin{cases} \frac{1}{1+\sum_{j=2}^{m} \exp(\mathbf{L}_i'\boldsymbol{\delta}_j + \gamma_j)} & c = 1 \\ \frac{\exp(\mathbf{L}_i'\boldsymbol{\delta}_c + \gamma_c)}{1+\sum_{j=2}^{m} \exp(\mathbf{L}_i'\boldsymbol{\delta}_j + \gamma_j)} & c \neq 1. \end{cases} \tag{1.9}$$

The MLE $\hat{\boldsymbol{\rho}}$ of $\boldsymbol{\rho}$ satisfies the following set of estimating equations

$$\frac{1}{n}\sum_{i=1}^{n} \begin{pmatrix} 1 \\ \mathbf{L}_i \end{pmatrix} \{I(C_i = c) - g(c, \mathbf{L}_i; \hat{\boldsymbol{\rho}})\} = \mathbf{0}, \qquad c = 2, \ldots, m. \tag{1.10}$$

First, we perform a Taylor expansion of (1.10) around $\boldsymbol{\rho}$ and obtain

$$\sqrt{n}(\hat{\boldsymbol{\rho}} - \boldsymbol{\rho}) = \frac{1}{\sqrt{n}}\sum_{i=1}^{n} A_1^{-1} R_{i1} + o_p(1), \tag{1.11}$$

where

$$R_{i1} = \begin{pmatrix} 1 \\ \mathbf{L}_i \end{pmatrix} \{I(C_i = c) - g(c, \mathbf{L}_i; \hat{\boldsymbol{\rho}})\},$$
$$A_1 = E\left(\frac{\partial R_{i1}}{\partial \boldsymbol{\rho}}\right).$$

Analogously, we perform a Taylor expansion of the outcome model estimating equations

$$\frac{1}{n}\sum_{i=1}^{n} \frac{\mathbf{X}_i}{g(C_i, \mathbf{L}_i; \hat{\boldsymbol{\rho}})} \left\{Y_i - U(\hat{\boldsymbol{\theta}}, \mathbf{X}_i)\right\} = \mathbf{0} \tag{1.12}$$

around $(\boldsymbol{\theta}', \boldsymbol{\rho}')'$ and obtain

$$\sqrt{n}(\hat{\boldsymbol{\theta}} - \boldsymbol{\theta}) = \frac{1}{\sqrt{n}}\sum_{i=1}^{n} A_2^{-1} \left\{R_{i2} + E\left(\frac{\partial R_{i2}}{\partial \boldsymbol{\rho}}\right)\sqrt{n}(\hat{\boldsymbol{\rho}} - \boldsymbol{\rho})\right\} + o_p(1), \tag{1.13}$$

where

$$R_{i2} = \frac{\mathbf{X}_i}{g(C_i, \mathbf{L}_i; \hat{\boldsymbol{\rho}})} \left\{Y_i - U(\hat{\boldsymbol{\theta}}, \mathbf{X}_i)\right\},$$
$$A_2 = E\left(\frac{\partial R_{i2}}{\partial \boldsymbol{\theta}}\right).$$

Now, let

$$U_c(\hat{\boldsymbol{\theta}}, \mathbf{X}_i) = \text{expit}\left(\mathbf{L}_i'\hat{\boldsymbol{\beta}} + \hat{\psi}_c\right),$$

then we define the estimator for the potential full population risk as follows:

$$\hat{E}\{Y(c)\} = \frac{1}{n}\sum_{i=1}^{n} V_c(\hat{\boldsymbol{\theta}}, \hat{\boldsymbol{\rho}}, \mathbf{X}_i) \tag{1.14}$$

$$= \frac{1}{n}\sum_{i=1}^{n} \left[U_c(\hat{\boldsymbol{\theta}}, \mathbf{X}_i) + \frac{I(C_i = c)}{g(c, \mathbf{L}_i; \hat{\boldsymbol{\rho}})} \left\{ Y_i - U_c(\hat{\boldsymbol{\theta}}, \mathbf{X}_i) \right\} \right] \tag{1.15}$$

$$= \frac{1}{n}\sum_{i=1}^{n} \left[\frac{I(C_i = c)Y_i}{g(c, \mathbf{L}_i; \hat{\boldsymbol{\rho}})} + \left\{ 1 - \frac{I(C_i = c)}{g(c, \mathbf{L}_i; \hat{\boldsymbol{\rho}})} \right\} U_c(\hat{\boldsymbol{\theta}}, \mathbf{X}_i) \right]. \tag{1.16}$$

Combining (1.11) and (1.13), the variance of the estimated potential full population risk $\hat{E}\{Y(c)\}$ on the risk scale is estimated via

$$\mathrm{Var}\left[\hat{E}\{Y(c)\}\right] = \frac{1}{n}\mathrm{Var}\left[V_c(\boldsymbol{\theta}, \boldsymbol{\rho}, \mathbf{X}_i) + E\left(\frac{\partial V_c}{\partial \rho}(\boldsymbol{\theta}, \boldsymbol{\rho}, \mathbf{X}_i) \right) A_1^{-1} R_{i1} \right.$$
$$\left. + E\left(\frac{\partial V_c}{\partial \theta}(\boldsymbol{\theta}, \boldsymbol{\rho}, \mathbf{X}_i) \right) A_2^{-1} \left\{ R_{i2} + E\left(\frac{\partial R_{i2}}{\partial \boldsymbol{\rho}} \right) A_1^{-1} R_{i1} \right\} \right]. \tag{1.17}$$

Again, the above expressions can be transformed to the logit scale and continue to hold when the parameters $\boldsymbol{\theta}$ are estimated according to Firth's modified estimating equations (1.2).

2. Additional results

An illustration of model extrapolation considering two centers with strongly differential case-mix is given in Figure 1.

2.1 *Simulation study application: Belgian Colorectal Cancer register*

The ME models were fitted in R using the `rjags` package (Plummer, 2003) where we assigned the following independent non-informative hyperpriors:

Classical normal ME model	*Clustered normal ME model*	
$\mu_\psi \sim N(0, 25)$	$\mu_k \sim N(0, 25)$	$k = 1, \ldots, K$
$\sigma_\psi \sim U(0, 10)$	$\sigma_k \sim U(0, 10)$	$k = 1, \ldots, K$
$\beta_j \sim N(0, 10^2)$	$\beta_j \sim N(0, 10^2)$	$j = 1, \ldots, \ell,$

where $K = 3$ denotes the number of center clusters and ℓ is the length of the vector of patient characteristics. The Firth corrected FE model was fitted in R using the `brglm` package (Kosmidis, 2011). The analysis of 5 simulated datasets took on average 3 hours for the classical normal ME method and 15 hours for the clustered analog, as compared to at most 3 minutes for the FE and doubly robust methods.

First we determine 'true' center classifications based on a large sample of simulated data for 10^6 patients in 63 centers. Next, we generate 1000 samples of each 2355 patients under the same models. In this data-generating process, we first generated independent patient characteristics age, gender and initial disease status cStage based on descriptive statistics in Goetghebeur *and others* (2011), such that age was 67 years on average (SD 9 years), that 61% of the patients were male and that 13%, 18%, 51%, 14% respectively had cStage I to IV; 4% had missing cStage. Next, we use the PS and outcome models that were fitted on the observed data as data-generating

models. So, the patients' center choice and outcome are generated, mimicking the center-specific distribution of patients in the register. Then, based on the large random sample of 10^6 patients we obtain precise estimates of $E(Y)$ and $E\{Y(c)\}$ as sample averages. Following equations (2.2) and (2.3) in the main text, this results in the true center classification. For each center this true classification is then compared to the classification based on a sample of $n = 2355$ patients and using one of the regression methods to estimate $\hat{E}(Y)$ and $\hat{E}\{Y(c)\}$. In the analysis, we excluded centers treating less than 10 patients, resulting in a median of 33 registered patients per center and an average of 53 centers.

About half of the centers are labelled as having low/high risk based on the initial large sample (see main text Figure 1, ignoring the simulation results for now) although for many centers the 'true' potential full population risk is close to the decision limits, especially on the low side. Note that results depend highly on the values of the clinical (λ) and statistical (k) tolerance levels. When early detection is most important, such as for confidential feedback to clinical centers, large power is preferred over small type I errors. In contrast, when publishing results openly, mistakenly classifying a center as high risk may have unduly severe implications for that center (decrease in number of patients, lower funding) compared to mistakenly failing to recognize a center as potentially high risk.

Tables 1 and 2 provide extra results on simulations modelled on the Belgian Colorectal Cancer register when the Firth correction is not applied and when the sample size is doubled. We performed additional simulations to investigate the effect of model misspecification. We generate data under an outcome regression model with quadratic centered age effect and a PS model with the absolute value of centered age effect. Misspecification is introduced in the fitted outcome and/or PS model by estimating a linear centered age effect instead. The original population average risk $E(Y) = 23\%$ based on Goetghebeur *and others* (2011) is retained. Center effects were multiplied by 2 to obtain a similar spread of $E\{Y(c)\}$ values compared to the original simulations. The original age coefficients in the PS models are multiplied by 2 to obtain more extreme differences in age distribution and thus stronger extrapolation across centers.

Descriptives under the new and original data generating models for a random sample of $n = 2355$ patients are respectively given in Figures 2 and 3, where centers with size smaller than 10 are indicated in red. The quadratic age effect is quite pronounced while the corresponding estimated linear age effect is approximately zero (See bottom of figure 2). Comparing the age distribution under both simulation scenarios, we see more overlap in the age distribution across centers under the new data generating PS model.

Simulation results in table 3 show that the Firth corrected fixed effects and doubly robust PS methods have similar performance when the regression models are correctly specified. We summarize the bias on $E\{Y(c)\}$ over all centers by taking the difference of the mean of the squared bias per center and the mean of the empirical variance of the bias over simulations and next taking the square root, that is

$$\sqrt{E_c\left[\text{Bias}(E\{Y(c)\})^2\right] - E_c\left[\frac{\text{Var}\left(\hat{E}\{Y(c)\}\right)}{S}\right]}, \tag{2.18}$$

where S is the number of simulations and E_c is the average over all centers $c = 1, \ldots, m$. Note that we correct for the fact that the squared bias also depends on the variance of the bias. In general the coverage of the 95% CI for $E\{Y(c)\}$ is smaller than 95%, but this may be due to extrapolation across centers, which is also reflected in the bias. For both methods the power to detect high mortality risk centers decreases when the outcome model is misspecified, but

the Type I errors do not decrease accordingly. Here, the doubly robust PS method outperforms the fixed effects method in terms of coverage of the 95% CI for $E\{Y(c)\}$, percentage of correct classification and bias on $E\{Y(c)\}$, although it does not gain power to detect high mortality risk centers. Misspecification of the PS model does not seem to have a large impact on the results in our simulation study, although this may be different for other data generating models. When both models are misspecified, the doubly robust PS method performs worse than the fixed effects method with misspecified outcome model.

2.2 *Analysis of the Swedish Stroke Register*

We give descriptive statistics for both the original and reduced dataset in Table 4. To assess the extent of differential patient case-mix across centers, we perform a univariate analysis on the complete cases (Figures 6 to 8). In general case-mix does not differ much across centers, but we detect considerable variation with respect to treatment for high blood pressure (Figure 4), education level (Figure 5) and time of admission (Figure 6). The minimum number of patients for whom we have records per hospital is 104 (median 1383.5 and max 7260) for the full data and 49 (median 957.5 and max 4341) for the complete cases. Some centers have no records for several years because they no longer treat stroke patients, have been merged with another center, or only started treating stroke patients during the study. There are no missing values for the outcome and the 30-day mortality risk is lower for the complete cases (8%) than for the full data (12%), which is due to the complete cases being generally more healthy as can be seen from the patient characteristics measured on admission (see Table 4). Figure 8 shows lower center-specific mortality risks for the complete cases compared to all cases, so results will probably be too optimistic for all centers. To describe the effect of risk factors on outcome, we report the estimated odds ratios based on fitting a logistic normal ME model (see Table 5).

The analysis is based on a logistic model for the outcome in which we allowed for a piecewise linear spline effect of standardized age (mean age 75 years, $SD = 12$ years) with a knot at age 80. Besides the main effects listed in Table 5, we include three interactions between patient-specific covariates which are of primary interest: education level by age exceeding 80 to account for a different effect of education level on 30-day mortality for elderly or young patients, institution by living alone to account for a different effect of living alone for patients in an institution or at home, and living alone by gender to account for a different effect of living alone for men and women. Note that we do not include interactions with center in the model. The time-dependent PS model includes the same risk factors, with exception of the interactions, and it does not allow for a piecewise linear effect of age to avoid model fitting problems. Results for the analysis of the Swedish Stroke Register when applying the Firth correction to the outcome model for the FE method and the doubly robust method are given in Figures 9 to 11.

3. R-code

Software in the form of R-code is available at
https://github.ugent.be/mmvarewy/Biostatistics2014.

References

Firth, D. (1992). Bias reduction, the Jeffreys prior and GLIM. In: Fahrmeir, Ed. L., Francis, B., Gilchrist, R. and Tutz, G. (editors), *Advances in GLIM and Statistical Modelling*. New York:

Springer-Verlag. pp. 91–100.

Firth, D. (1993). Bias reduction of maximum likelihood estimates. *Biometrika* **80**(1), 27–38.

Goetghebeur, E., Van Rossem, R., Baert, K., Vanhoutte, K., Boterberg, T., Demetter, P., De Ridder, M., Harrington, D., Peeters, M., Storme, G., Verhulst, J., Vlayen, J., Vrijens, F., Vansteelandt, S. *and others*. (2011). Quality insurance of rectal cancer - phase 3: statistical methods to benchmark centers on a set of quality indicators, Good Clinical Practice (GCP). *Belgian Health Care Knowledge Centre (KCE)* **KCE Report 161C, D/2011/10.273/40**, 1–142.

Heinze, Georg. (2006). A comparative investigation of methods for logistic regression with separated or nearly separated data. *Statistics in medicine* **25**(24), 4216–4226.

Kessels, R., Jones, B. and Goos, P. (2013, August). An argument for preferring firth bias-adjusted estimates in aggregate and individual-level discrete choice modeling. *Technical Report* D/2013/1169/013, University of Antwerp.

Kosmidis, I. (2011). brglm: Bias reduction in generalized linear models.

Plummer, M. (2003). Jags: A program for analysis of bayesian graphical models using gibbs sampling. In: *Proceedings of the 3rd International Workshop on Distributed Statistical Computing (DSC 2003). March*. pp. 20–22.

	Fixed effects	Doubly robust PS
Power (%)		
to detect High	59.6	52.6
to detect Low	38.5	45.3
Type I error (%)		
Low as High	1.8	5.5
Accepted as High	7.4	10.5
Type I error (%)		
High as Low	1.5	4.2
Accepted as Low	14.9	23.9
Coverage of 95% CI		
for $E\{Y(c)\}$ (%)	94.4	88.5
Classification		
% High (22%)	15.3	16.9
% Low (30%)	19.6	25.9
% correct (L-A-H)	62.5	57.6

Table 1. Center classification (Low/High risk or Accepted) based on 1000 simulations for FE and doubly robust PS method without Firth correction. The true percentage of low and high risk centers is respectively 30% and 22%.

	Classical normal ME	Clustered normal ME	Firth corrected FE	Firth corrected doubly robust PS
Power (%)				
to detect High	41.2	40.3	60.6	55.1
to detect Low	22.8	13.4	36.1	39.7
Type I error (%)				
Low as High	0.1	0.2	1.2	3.5
Accepted as High	1.0	1.8	6.4	9.4
Type I error (%)				
High as Low	0.2	0.2	0.6	1.8
Accepted as Low	4.1	4.4	10.1	16.6
Coverage of 95% CI				
for $E\{Y(c)\}$ (%)	95.8	84.4	94.8	90.6
Classification				
% High (22%)	9.5	9.8	16.9	17.7
% Low (30%)	9.2	6.5	16.7	21.2
% correct (L-A-H)	62.7	59.0	66.6	61.8

Table 2. Center classification (Low/High risk or Accepted) based on 1000 simulations, except for the Bayesian normal ME analyses which were evaluated on 100 simulations, with double sample size. The true percentage of low and high risk centers is respectively 30% and 22%.

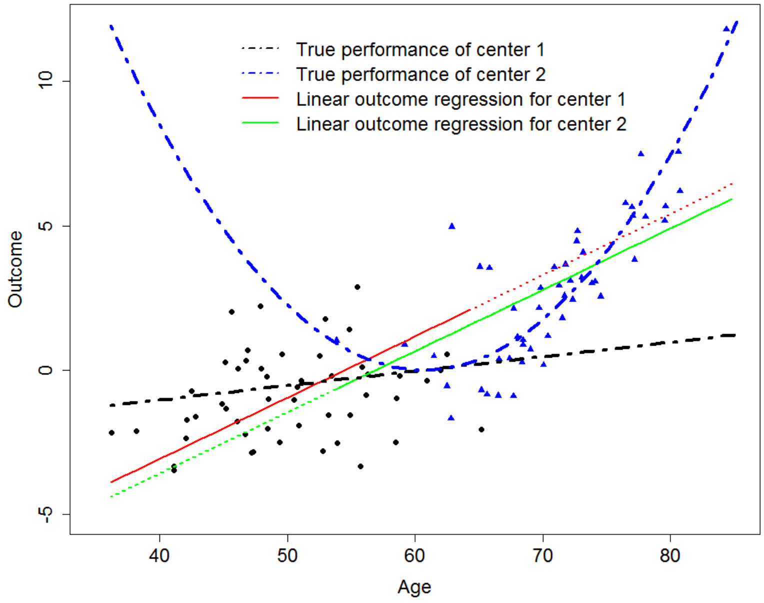

Fig. 1. Artificial example illustrating extrapolation under outcome regression with main effects for age and center. It visualizes outcome data for two centers with strongly differential case-mix and such that smaller outcomes are seen in center 1 (black dots) than in center 2 (blue triangles) in each patient subgroup. Direct standardization under seemingly well fitting linear regression models involves strong extrapolation (dashed line) of the outcome distribution in the given center to what it is expected to achieve on the full patient population under study. In truth, the observed data carry no information about $E\{Y(2)\}$ because no patients in center 2 have age below 60.

	Both models correct		Misspecification of			
			Outcome model		PS model	Both models
	FE	DR	FE	DR	DR	DR
Power (%)						
to detect High	65.4	64.7	54.2	50.1	64.8	50.2
to detect Low	41.6	43.0	40.1	41.8	40.4	47.4
Type I error (%)						
Low as High	1.5	4.5	5.3	5.0	4.7	7.0
Accepted as High	5.9	11.0	10.5	10.0	11.6	14.2
Type I error (%)						
High as Low	0.7	0.8	1.6	1.6	0.6	5.0
Accepted as Low	11.3	13.5	20.2	14.9	13.6	26.8
Classification						
% High (19%)	14.8	15.8	18.3	13.4	15.9	18.8
% Low (19%)	15.9	17.7	20.7	16.8	17.7	25.7
% correct (L-A-H)	72.9	70.1	60.3	66.2	70.0	56.8
Bias on $E\{Y(c)\}$ $(\times 10^{-2})$	1.54	3.62	5.85	5.63	3.52	8.32
MSE of $E\{Y(c)\}$ $(\times 10^{-2})$	0.330	0.447	0.922	1.01	0.458	1.32
For 95% *CI of* $E\{Y(c)\}$						
coverage (%)	88.7	83.2	84.2	90.2	84.2	78.6
median length	0.167	0.167	0.288	0.323	0.167	0.299

Table 3. Center classification (Low/High risk or Accepted) based on 1000 simulations, under model misspecification. FE = Fixed effects method, DR = Doubly robust PS method, both with Firth correction. The true percentage of low and high risk centers is 19%.

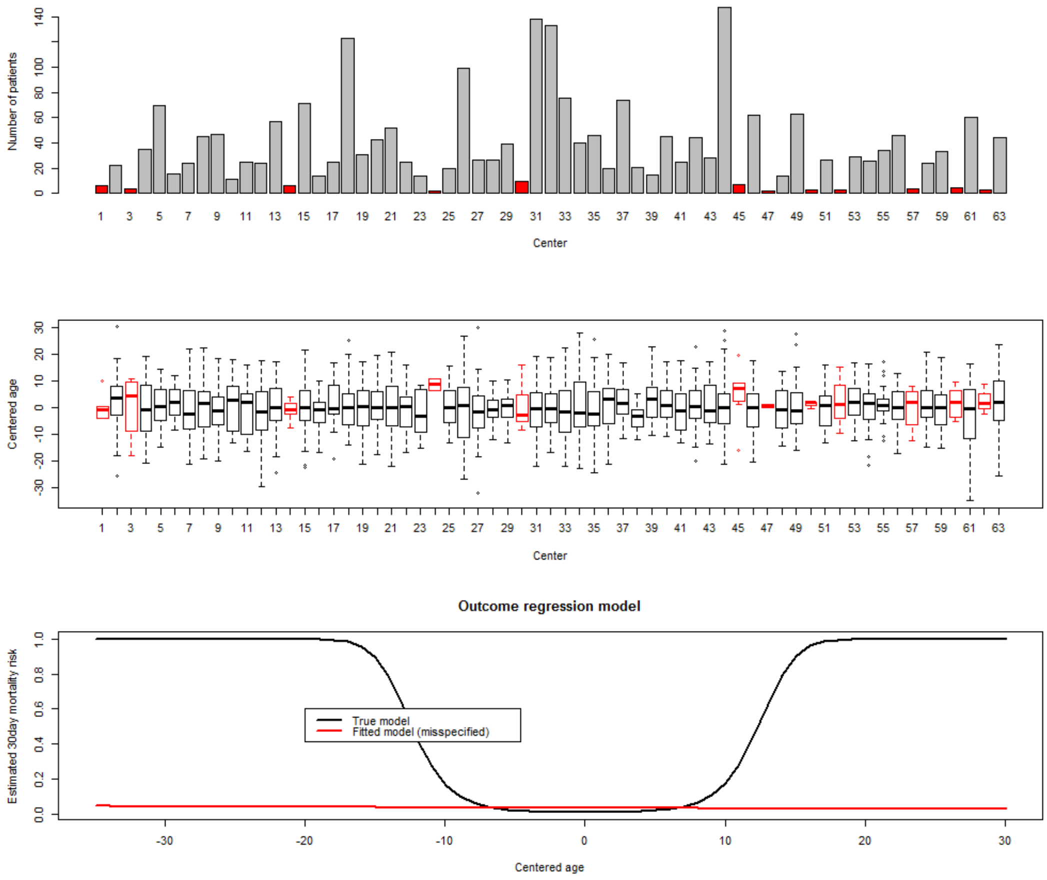

Fig. 2. Age distribution under the new data generating PS model (model misspecification), for a random sample of n = 2355 patients. Figure at the bottom: Fitted misspecified outcome model is a Firth corrected fixed effects model and estimated risks are for female patients with cStageI.

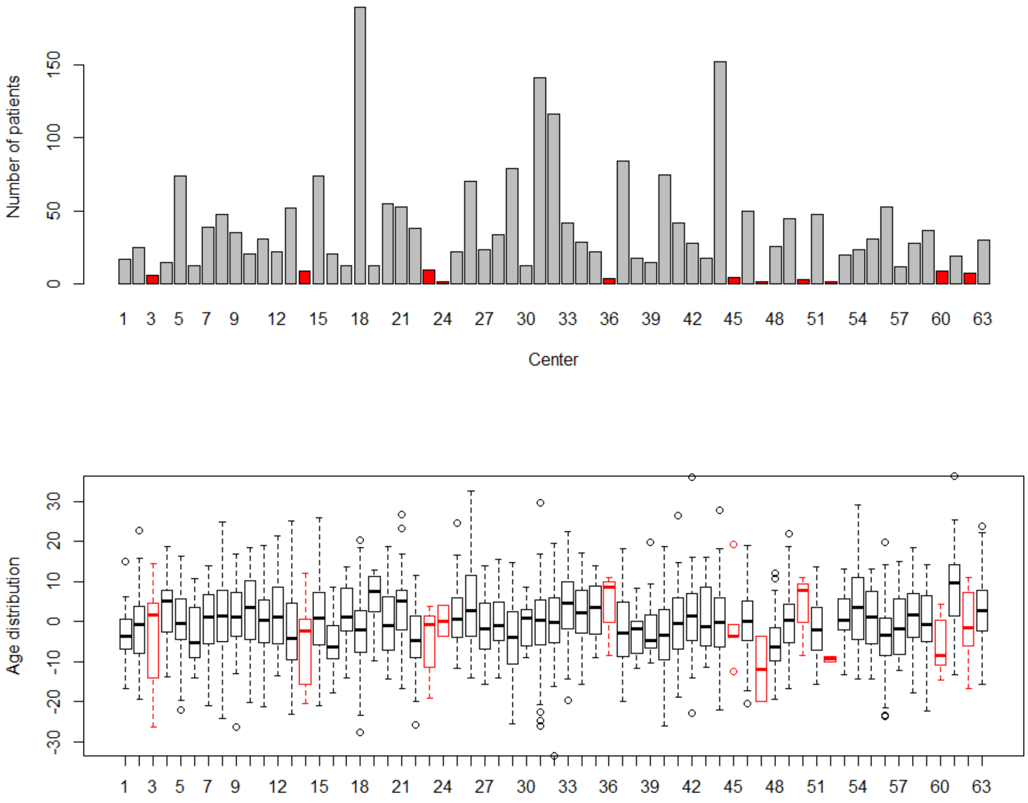

Fig. 3. Age distribution under the data generating PS model based on the Belgian colorectal cancer register.

Risk Factor	Full data Prevalence (%)	Missing (%)	Complete cases Prevalence (%)
Male	50.3	0	53.5
p-ADL dependence (*)	7.2	1.3	4.9
Institutional living (*)	7.0	0.5	4.0
Living alone (*)	48.9	0.7	42.7
Atrial fibrilation (*)	25.3	1.2	21.4
Diabetes (*)	18.9	0.5	19.1
Trt for high blood pressure (*)	50.3	1.4	50.3
Current smoker (*)	16.2	12.8	18.8
Stroke subtype		0	
(Intracerebral haemorrhage (I61))	12.1		11.8
Cerebral infarction (I63)	83.6		84.8
Unspecified stroke (I64)	4.3		3.4
Consciousness at admission		0.8	
(Alert)	82.9		86.8
Drowsy	12.1		9.7
Unconscious	5.0		3.5
Education		20.8	
(Primary)	51.9		51.6
Secondary	34.3		34.5
University	13.7		13.8
Adjusted yearly income (in 100 SEK)		0.5	
(< 185)	10.0		9.4
815 to 1235	39.0		34.0
1235 to 2241	40.4		44.1
> 2241	10.6		12.5
Year of admission		0	
(2001)	9.5		7.4
2002	10.3		8.7
2003	10.8		9.4
2004	11.1		10.6
2005	11.8		11.8
2006	11.6		12.0
2007	11.4		12.2
2008	11.7		13.0
2009	11.8		14.9

Table 4. Descriptive percentages of the binary and categorical covariates from the Swedish Stroke Register, indicating (*) if measured prior to stroke and (reference category) for analysis. For the full data we calculated percentages for each risk factor not taking into account missing values for that factor.

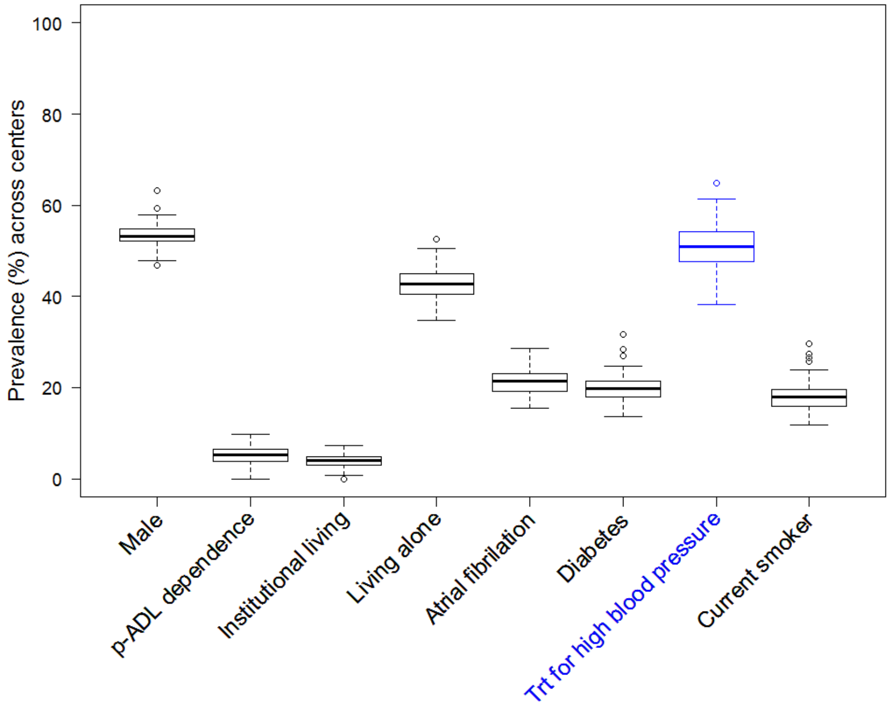

Fig. 4. Prevalence of the binary patient-specific covariates across centers based on the complete cases in the Swedish Stroke Register.

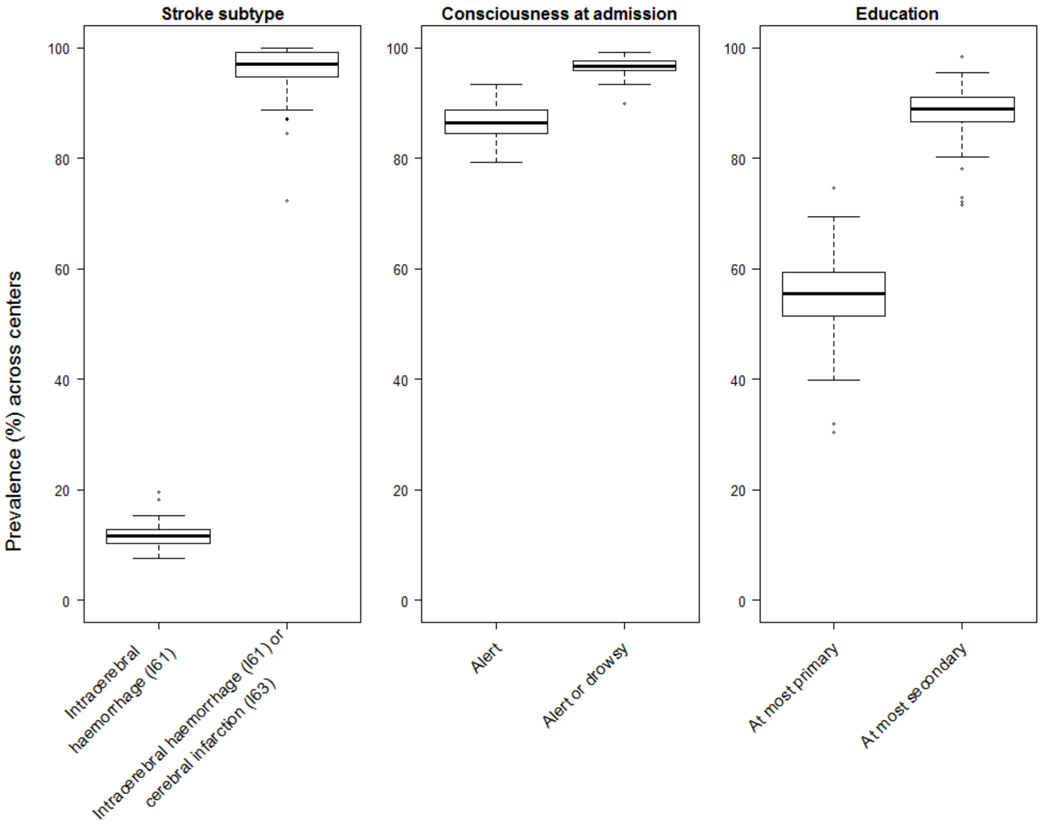

Fig. 5. Prevalence of stroke subtype, consciousness at admission and education across centers based on the complete cases in the Swedish Stroke Register.

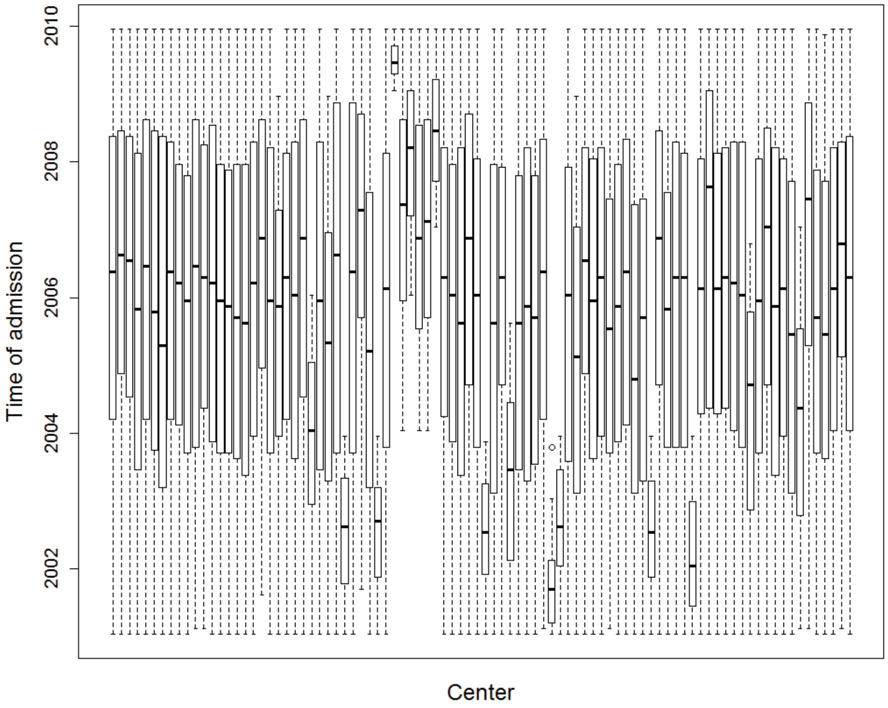

Fig. 6. Boxplot for the distribution of time of patient admission across centers based on the complete cases in the Swedish Stroke Register.

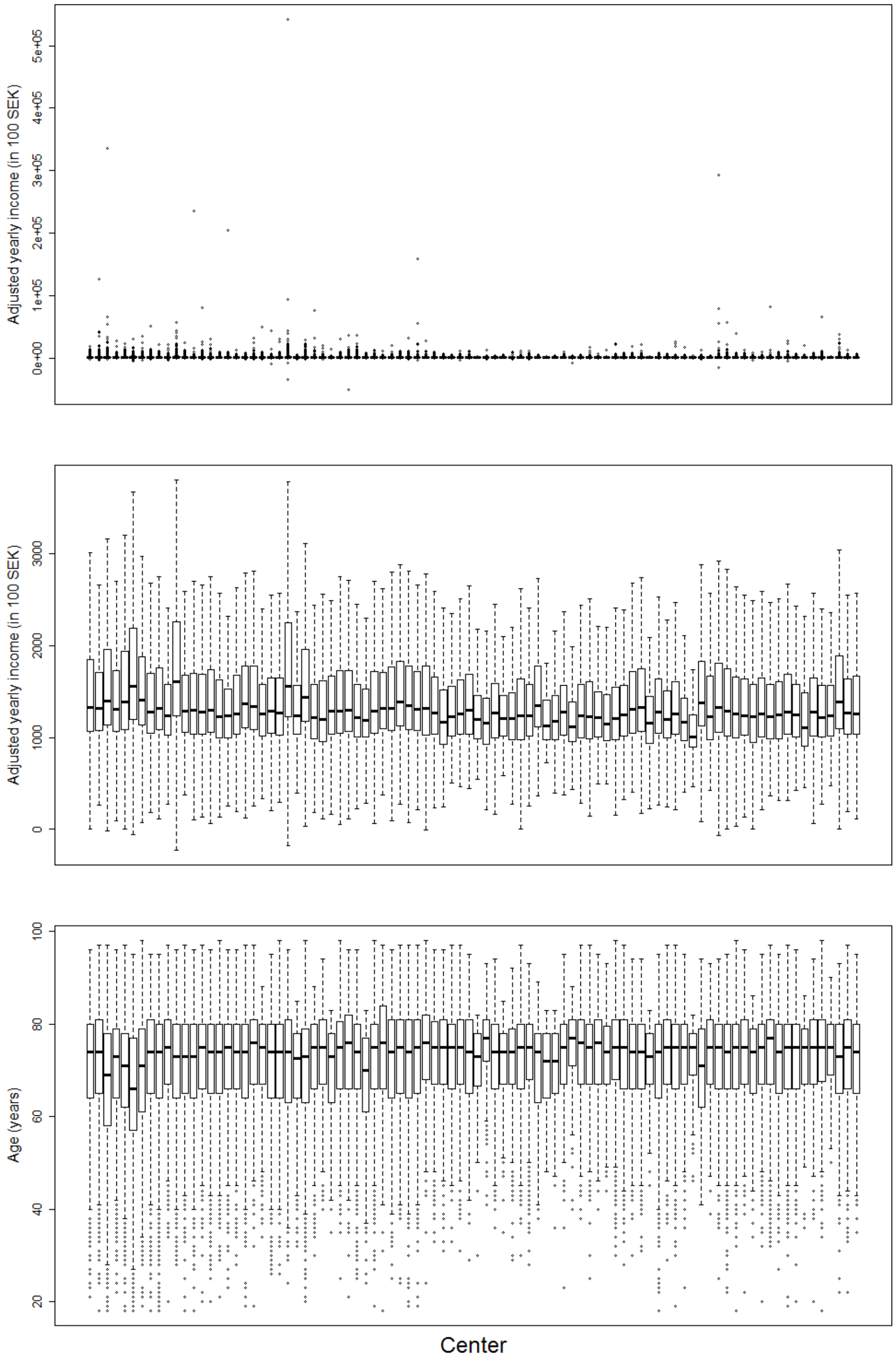

Fig. 7. Boxplots for the distribution of adjusted yearly income (in 100 SEK) with/without outliers and age per center based on the complete cases in the Swedish Stroke Register.

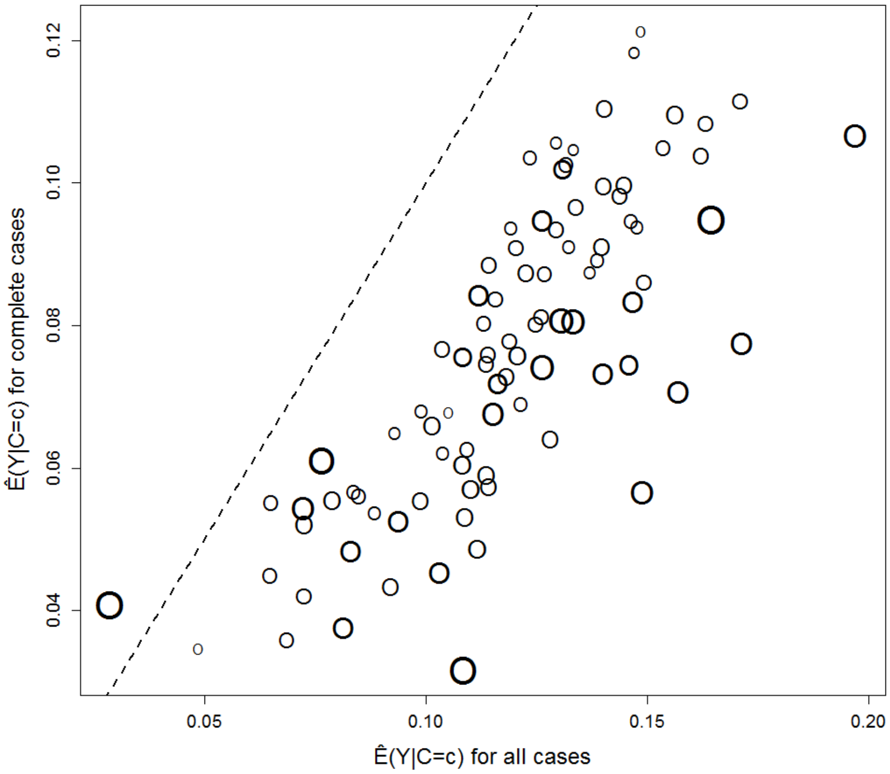

Fig. 8. Observed mortality risk per center for all cases and complete cases in the Swedish Stroke Register. The dashed line represents the first bisector and bulb sizes are proportional to the percentage of patients with missing values in that center.

Fixed effects	$\hat{\beta}$	SE($\hat{\beta}$)	$\hat{OR}$	95% CI for OR			p-value
(Intercept)	−2.854	0.084	0.058	0.049	to	0.068	< 0.001
Male	0.163	0.040	1.178	1.088	to	1.275	< 0.001
Age (standardized)	0.513	0.024	1.671	1.594	to	1.751	< 0.001
Age > 80 years	0.441	0.089	1.555	1.306	to	1.851	< 0.001
p-ADL dependence (*)	0.625	0.050	1.869	1.693	to	2.063	< 0.001
Institutional living (*)	0.533	0.123	1.703	1.337	to	2.169	< 0.001
Living alone (*)	0.020	0.043	1.020	0.938	to	1.109	0.639
Atrial fibrilation (*)	0.444	0.030	1.559	1.469	to	1.654	< 0.001
Diabetes (*)	0.205	0.034	1.227	1.149	to	1.311	< 0.001
Trt for high blood pressure (*)	−0.026	0.028	0.974	0.922	to	1.030	0.355
Current smoker (*)	0.114	0.040	1.121	1.035	to	1.213	0.005
Stroke subtype							
(vs Intracerebral haemorrhage (I61))							
Cerebral infarction (I63)	−0.933	0.034	0.393	0.368	to	0.421	< 0.001
Unspecified stroke (I64)	−0.419	0.072	0.657	0.571	to	0.757	< 0.001
Consciousness at admission (vs Alert)							
Drowsy	1.885	0.031	6.589	6.202	to	7.001	< 0.001
Unconscious	3.445	0.043	31.340	28.827	to	34.072	< 0.001
Education (vs Primary)							
Secondary	−0.094	0.035	0.910	0.849	to	0.976	0.008
University	−0.146	0.053	0.864	0.779	to	0.958	0.005
Adjusted yearly income (in 100 SEK)							
(vs < 185)							
815 to 1235	0.043	0.049	1.044	0.948	to	1.149	0.381
1235 to 2241	−0.012	0.049	0.988	0.898	to	1.088	0.811
> 2241	−0.146	0.065	0.864	0.760	to	0.982	0.025
Year of admission (vs 2001)							
2002	−0.071	0.074	0.931	0.806	to	1.076	0.335
2003	−0.025	0.071	0.975	0.848	to	1.122	0.726
2004	0.081	0.069	1.085	0.948	to	1.241	0.238
2005	0.115	0.068	1.122	0.983	to	1.281	0.089
2006	0.108	0.067	1.114	0.976	to	1.272	0.108
2007	0.104	0.067	1.109	0.972	to	1.266	0.123
2008	0.091	0.067	1.095	0.960	to	1.249	0.177
2009	0.097	0.067	1.101	0.965	to	1.257	0.151
Male × Living alone	−0.022	0.057	0.978	0.875	to	1.094	0.701
Institutional living × Living alone	0.030	0.131	1.031	0.797	to	1.333	0.816
Education (Secondary vs Primary)							
× Age > 80 years	0.050	0.135	1.051	0.807	to	1.368	0.711
Education (University vs Primary)							
× Age > 80 years	−0.036	0.213	0.965	0.635	to	1.466	0.867
**Random effects**	$\hat{\text{Var}}$						
Center	0.069						

Table 5. Model parameters for a descriptive logistic normal ME model with outcome 30-day mortality based on the Swedish Stroke Register, indicating (*) if measured prior to stroke and (vs reference category) for categorical covariates. SE = standard error, OR = odds ratio = $\exp(\beta)$, 95% CI for OR = 95% confidence interval for odds ratio.

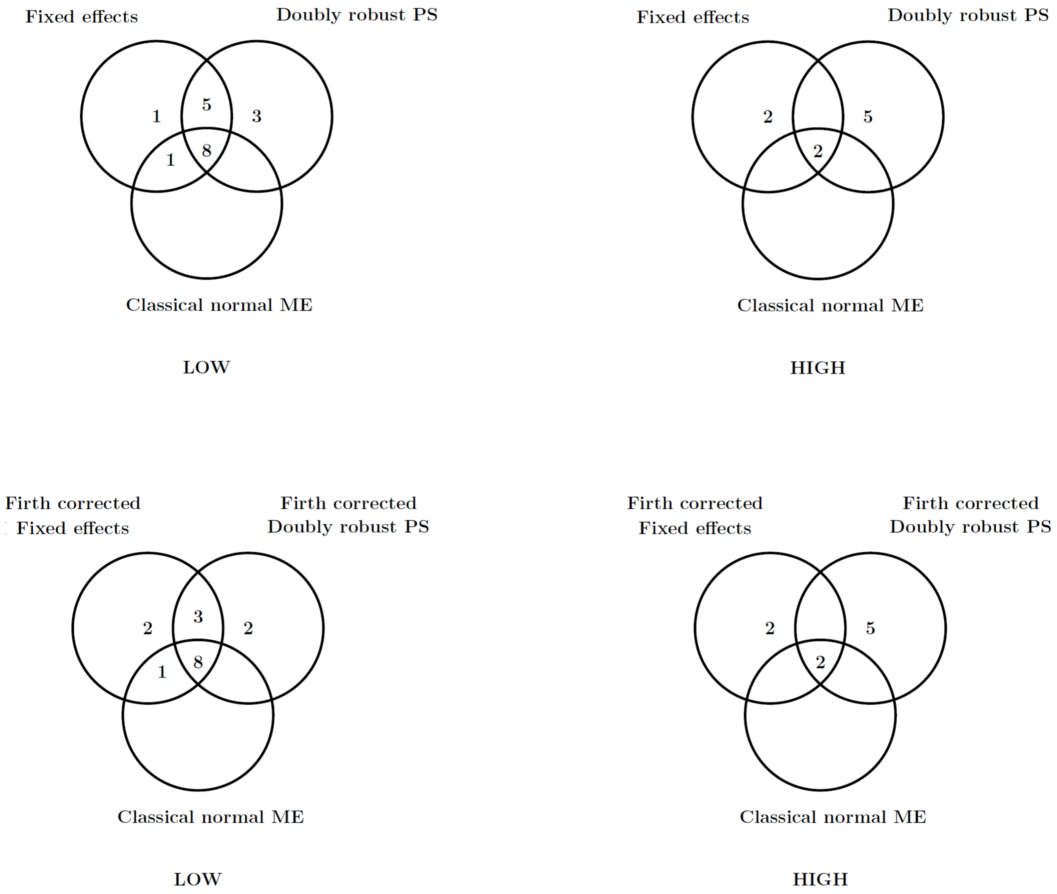

Fig. 9. Center classification for the 90 centers in the Swedish Stroke Register, without and with Firth correction.

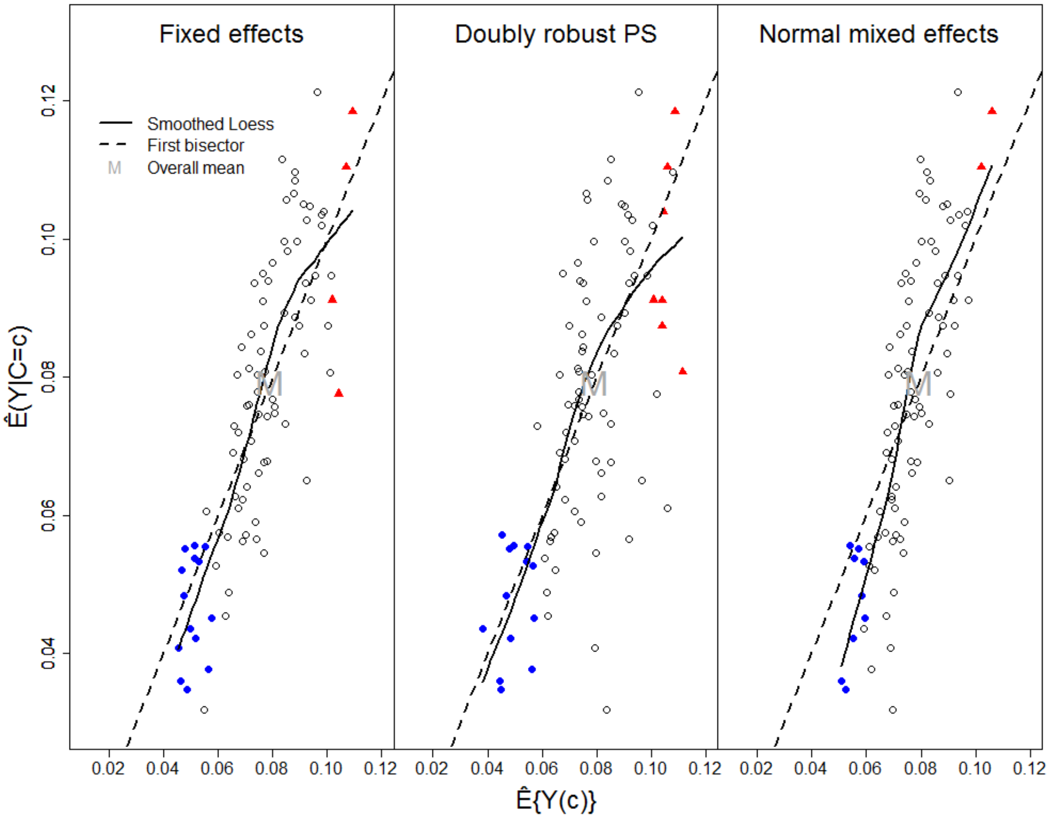

Fig. 10. The observed center-specific risk versus the potential full population risk for all 90 centers of Riks-Stroke for the FE method, the doubly robust PS method and the classical normal ME method, distinguishing between low (blue filled circles) and high (red triangles) mortality risk (with Firth correction).

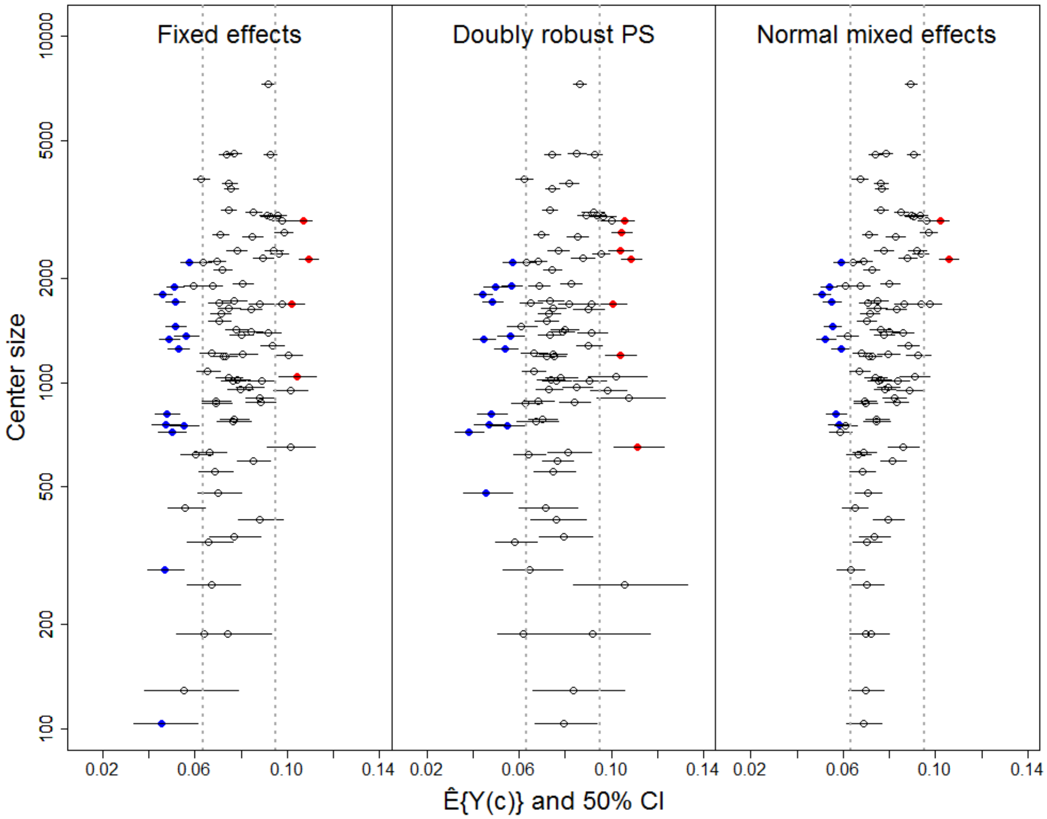

Fig. 11. The potential full population risk and corresponding 50% confidence interval for all 90 centers of Riks-Stroke for the FE method, the doubly robust PS method and the classical normal ME method. The vertical gray lines indicate the clinical decision limits $(1-\lambda)\hat{E}(Y)$ and $(1+\lambda)\hat{E}(Y)$, with $\lambda = 0.20$, which are used for classification of low (blue filled circles) and high (red filled circles) mortality risk centers (with Firth correction).
